# Protective effect of cannabinoid type 2 receptor agonist (JWH‐133) against hepatotoxicity induced by *Prangos ferulacea* Lindl. extract

**DOI:** 10.1113/EP094039

**Published:** 2026-09-12

**Authors:** Mesut Çelik, Aslı Çetin Taşlıdere, Zeynal Mete Karaca, Halil Düzova, İbrahim Halil Geçibesler, Ali Rıza Çalişkan, Çağatay Taşkapan, Kübranur Korkmaz

**Affiliations:** ^1^ Department of Physiology School of Medicine Inonu University Malatya Turkey; ^2^ Elderly Care Program, Vocational School of Health Services Bingöl University Bingöl Turkey; ^3^ Department of Histology and Embryology School of Medicine Inonu University Malatya Turkey; ^4^ Department of Medical Biology and Genetics School of Medicine Inonu University Malatya Turkey; ^5^ Department of Occupational Health and Safety School of Health Sciences Bingol University Bingöl Turkey; ^6^ Department of Gastroenterology, School of Medicine Inonu University Malatya Turkey; ^7^ Department of Medical Biochemistry School of Medicine Inonu University Malatya Turkey; ^8^ Medical Laboratory Techniques Program Vocational School of Health Services Malatya Turgut Ozal University Malatya Turkey

**Keywords:** cannabinoid type 2 receptor agonist, caspase‐3, endoplasmic reticulum stress hepatotoxicity, *Prangos ferulacea* Lindl

## Abstract

This study investigated *Prangos ferulacea* (PF)‐induced hepatotoxicity, its potential association with endoplasmic reticulum (ER) stress and proinflammatory cytokines, and the effect of the cannabinoid type 2 receptor agonist JWH‐133. Rats (*n* = 37) were divided into four groups: control–sham group (CSG, *n* = 9), PF extract group (PG, *n* = 9), PF extract + JWH‐133 group (PJG, *n* = 9) and JWH‐133 group (JG, *n* = 10). Hepatic ER stress markers (CHOP, GRP78 and ATF4), interleukin (IL)‐17, IL‐23, heat shock protein 72 (HSP72), and calpain, as well as serum IL‐17, IL‐23, aspartate aminotransferase (AST), alanine aminotransferase (ALT), total protein and albumin, were assessed. Liver histology was evaluated using haematoxylin–eosin staining, and apoptosis was assessed by caspase‐3 immunohistochemistry. No significant between‐group differences were observed in hepatic GRP78, CHOP, ATF4, IL‐17 or IL‐23 levels. Similarly, serum IL‐17 and IL‐23 levels did not differ significantly among the groups. Calpain levels differed significantly among the groups (*P =* 0.0017), with higher levels in PG (*P =* 0.0325) and PJG (*P =* 0.0076) than in CSG. Compared with CSG, PG and PJG showed lower ALT (*P =* 0.0021 for both), AST (*P =* 0.0021 for both), albumin (*P =* 0.0031 and *P =* 0.0016, respectively) and total protein (*P =* 0.0056 and *P =* 0.0017, respectively) levels. Histopathological damage was significantly greater in PG than in CSG, PJG and JG (all *P <* 0.0001). Caspase‐3 immunoreactivity was intense in PG and limited in PJG. PF extract caused marked liver injury without clear canonical ER stress activation. JWH‐133 attenuated histopathological damage, consistent with the potential role of CB2 receptor activation. These findings support ER stress‐independent mechanisms and warrant further investigation of CB2 activation as a hepatoprotective strategy.

## Abstract (highlights)

- **What is the central question of this study?**
  Does *Prangos ferulacea* extract induce hepatotoxicity through endoplasmic reticulum stress and proinflammatory cytokines, and if so can the cannabinoid type 2 receptor agonist JWH‐133 attenuate this damage?
- **What is the main finding and its importance?**
*Prangos ferulacea* extract caused marked histopathological liver injury, reduced hepatic synthetic capacity, and increased caspase‐3‐mediated apoptosis without activating canonical endoplasmic reticulum stress markers (CHOP, GRP78, ATF4) or the IL‐17–IL‐23 axis. The cannabinoid type 2 receptor agonist JWH‐133 substantially attenuated this damage, supporting CB2 receptor activation as a candidate hepatoprotective strategy in plant‐induced liver injury and indicating that the protective effect operates via endoplasmic reticulum stress‐independent mechanisms.

## 1 INTRODUCTION

*Prangos ferulacea* Lindl. (PF), a member of the Apiaceae family, is used for various purposes in different cultures. The plant is consumed as a food in several cuisines and has also been traditionally used for its sedative, anti‐inflammatory, antiviral, antifungal and antibacterial properties, as well as for the treatment of gastrointestinal disorders (Asadi‐Samani et al., 2015). Although most reports have emphasized the beneficial and protective properties of PF, toxicological studies have also raised concerns regarding the safety of PF‐derived constituents such as osthole (Shokoohinia et al., 2017). In addition, acute toxic hepatitis following *P. ferulacea* consumption has been reported clinically (Dursun et al., 2019). These clinical observations raise concerns regarding the potential hepatotoxic effects of PF and highlight the need to elucidate the underlying molecular mechanisms.

The liver is particularly vulnerable to toxic insults, and hepatotoxic stimuli can activate several interconnected intracellular stress responses, including oxidative stress, mitochondrial dysfunction, lysosomal damage, inflammation and endoplasmic reticulum (ER) stress (Malhi & Kaufman, 2011). The ER is essential for protein folding, maturation and cellular homeostasis and is tightly regulated by protein oxidoreductases, molecular chaperones, proteolytic systems, glycosylation and sulfation enzymes, and Ca^2^^+^ transporters. Various physiological and pathological conditions, including B‐cell maturation, insulin secretion, nutrient deprivation, oxidative stress, hypoxia and mutations in ER‐related genes, can disrupt ER homeostasis and consequently induce ER stress (Ajoolabady et al., 2023; Szegezdi et al., 2006). When ER homeostasis is disturbed, cells activate adaptive mechanisms to restore protein homeostasis; however, persistent or severe ER stress may contribute to cellular injury and apoptosis.

Heat shock proteins (HSPs), particularly HSP72, are important components of the cellular response to stress and play a critical role in maintaining protein homeostasis. These molecular chaperones facilitate the correct folding of newly synthesized or damaged proteins and prevent the accumulation of misfolded proteins, thereby contributing to cellular survival under stressful conditions (Asea, 2007; Walsh et al., 2001). In addition to chaperone‐mediated responses, Ca^2^^+^‐dependent signaling pathways may contribute to ER stress‐associated cell death. Calpains are a family of Ca^2^^+^‐dependent cysteine proteases that cleave a wide range of intracellular protein substrates and have been implicated in apoptosis. In hepatic stellate cells, calpain‐2 has been reported to promote apoptosis through a Ca^2^^+^‐dependent/caspase‐12‐mediated ER stress pathway (Liu et al., 2021). Therefore, alterations in HSP72 and calpain‐related pathways may provide important insights into the cellular mechanisms underlying toxin‐induced hepatic injury.

Inflammatory responses are another important component of liver injury. Interleukin (IL)‐23 is a proinflammatory cytokine closely associated with the differentiation and maintenance of Th17 cells and is functionally linked to IL‐17, another important proinflammatory cytokine predominantly produced by activated T cells. The IL‐23–IL‐17 axis plays a significant role in the regulation of inflammatory responses, and increased levels of these cytokines may reflect and contribute to tissue injury (Iwakura & Ishigame, 2006). Hepatotoxicity induced by plant‐derived compounds may involve several interconnected pathogenic mechanisms, including apoptosis, ER stress, cytochrome P450‐mediated bioactivation, oxidative stress and mitochondrial dysfunction (Stedman, 2002). As the severity of hepatic injury increases, inflammatory and immune responses may consequently be activated, resulting in elevated levels of proinflammatory cytokines such as IL‐17 and IL‐23 (Li et al., 2021). Thus, evaluation of these cytokines may provide additional evidence regarding the inflammatory component of PF‐induced hepatotoxicity.

The endocannabinoid system regulates various physiological functions in mammals, including pain, inflammation, appetite and psychoactive responses. The system exerts these effects through multiple receptors and receptor‐mediated pathways. Various natural or synthetic substances bind to cannabinoid type 1 (CB1) and cannabinoid type 2 (CB2) receptors in the endocannabinoid system. CB1 receptors are predominantly expressed in the nervous system (Tutun & Baydan, 2018), and CB2 receptors are primarily expressed in immune cells, the spleen, tonsils, testes and hepatic myofibroblasts. They play important roles in immune regulation in the tissues where they are expressed (Cabral et al., 2008). Recent studies have shown that activation of CB2 receptors expressed on fibrogenic cells may limit liver damage during acute liver injury by reducing fibrogenic activity. A CB2 receptor agonist has been shown to reduce hepatocyte apoptosis and increase liver regeneration (Teixeira‐Clerc et al., 2010).

Although previous studies have predominantly reported beneficial or protective effects of PF, seven patients from the Hakkari region of Türkiye who developed PF‐associated hepatotoxicity were referred to Turgut Özal Medical Center. This clinical observation, together with the limited number of experimental studies investigating the hepatotoxic potential and underlying mechanisms of PF, emphasizes the need for further investigation.

Therefore, the present study aimed to investigate whether PF extract induces hepatotoxicity and to elucidate its potential association with ER stress, apoptosis‐related pathways and proinflammatory cytokines. In addition, we evaluated whether pharmacological activation of the cannabinoid type 2 receptor with the selective CB2 agonist JWH‐133 could attenuate PF‐induced hepatic injury. By examining these pathways together, this study sought to provide further insight into the mechanisms underlying PF‐associated hepatotoxicity and to determine the potential protective role of CB2 receptor activation.

## 2 METHODS

### 2.1 Ethical approval

All experimental procedures were approved by the Inonu University Faculty of Medicine Experimental Animals Ethics Committee (approval no. 2019/A‐34) and were conducted in accordance with the relevant Turkish national legislation governing the care and use of laboratory animals, as well as the ethical principles and animal experimentation policies under which *Experimental Physiology* operates.

### 2.2 Animals

The animals were housed at 20–22°C under a 12‐h light–dark cycle. They had ad libitum access to standard rat chow and tap water. Experimental procedures were conducted at the Inonu University Faculty of Medicine Experimental Animal Production and Research Center, from which the animals were also obtained. Male Wistar albino rats aged 8–10 weeks were used in the study (*n* = 40). The animals were weighed before and after the experiment. The sample size was determined a priori using G*Power 3.1 (Faul et al., 2007) based on previously reported effect sizes for histopathological liver injury in rat hepatotoxicity studies (Tutun & Baydan, 2018). With α = 0.05, a power of 0.80, and an estimated large effect size (*f* = 0.50) for between‐group comparisons, a minimum of 8–10 animals per group was required; accordingly, 9–10 animals were allocated to each group. Group allocation was performed using a computer‐generated random sequence, and biochemical and histopathological analyses were performed by investigators blinded to group assignment.

### 2.3 Experimental groups

Animals were randomly assigned to four groups:

Group 1: vehicle solution, control–sham group (CSG, *n* = 9);
Group 2: PF hydroalcoholic extract (PFE) group (PG, *n* = 9);
Group 3: PFE + cannabinoid type receptor agonist (JWH‐133) group (PJG, *n* = 9);
Group 4: cannabinoid type 2 receptor agonist (JWH‐133) group (JG, *n* = 10).

During the experimental period, three animals died (two from PG and one from PJG). These deaths occurred early in the experimental protocol, between days 2 and 3 of dosing, and were attributed to acute toxic effects of the PF extract based on macroscopic post‐mortem examination. To maintain equal group sizes, one animal originally assigned to the control group was reassigned to PG on day 3 of the experiment, before any biochemical or histological data had been collected. Before reassignment, the animal had received only the vehicle solution and no active treatment (PF or JWH‐133); from that time point onward, it received the remaining PF dosing schedule. The reassignment occurred before any outcome data were collected and was not based on biochemical or histological results; the animal was included in PG for all subsequent analyses.

### 2.4 Preparation of *Prangos ferulacea* Lindl. extract

The aerial parts of *Prangos ferulacea* (L.) Lindl. (Apiaceae) were collected during the flowering stage from the Hakkari region of southeastern Türkiye (37.5744°N, 43.7408°E; approximately 1700 m altitude). The plant material was authenticated by a botanist at the Department of Pharmaceutical Botany, Faculty of Pharmacy, Inonu University. The taxonomic identity of the collected specimens was confirmed as *P. ferulacea* (L.) Lindl. in accordance with the World Flora Online database (www.worldfloraonline.org). Voucher specimens were prepared and deposited in the herbarium following appropriate drying procedures.

The collected aerial parts were cut into suitable pieces and extracted with ethyl alcohol (Sigma‐Aldrich, St Louis, MO, USA). The resulting extract was filtered and concentrated under reduced pressure using a rotary evaporator (Heidolph Rotary Evaporator, Schwabach, Germany) to remove the solvent. The extraction procedure was performed in the Department of Occupational Health and Safety at Bingol University. The resulting crude extract was stored at 4°C until further use. (Farkhad et al., 2012).

### 2.5 Experimental procedure

Before initiation of the experiment, all animals underwent a 1‐week acclimatization period. Body weights were recorded at baseline, prior to the start of the experimental protocol, and again at the end of the study. During the 14‐day experimental period, rats in the control‐sham group (CSG) received 1 mL of vehicle solution intraperitoneally (i.p.), consisting of 95% phosphate‐buffered saline (PBS) and 5% dimethyl sulfoxide (DMSO). Rats in the PF group (PG) were administered PF extract intraperitoneally at a dose of 250 mg/kg/day for 14 consecutive days. The PF dose of 250 mg/kg/day was selected based on previously published rodent studies investigating the hepatotoxic and pharmacological effects of PF and related Apiaceae extracts (Farkhad et al., 2012; Farokhi et al., 2012; Mokhtari & Mohammadi, 2012).

**FIGURE 1:**
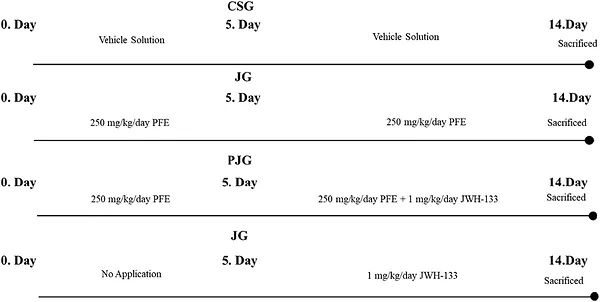
Timeline of experimental procedures in rats.

In the PF + JWH‐133 group (PJG), PF extract dissolved in 6% physiological saline was administered intraperitoneally at a dose of 250 mg/kg/day for 14 days. Beginning on day 6, JWH‐133 (Axon Medchem, Virginia, USA), dissolved in DMSO (Sigma‐Aldrich) and diluted with 99% phosphate buffer, was additionally administered intraperitoneally at a dose of 1 mg/kg/day for 9 consecutive days. In the JWH‐133 group (JG), rats received JWH‐133 intraperitoneally at a dose of 1 mg/kg/day from day 6 to day 14. For this group, JWH‐133 was dissolved in 5% DMSO and diluted with 95% phosphate buffer. The JWH‐133 dose of 1 mg/kg/day was selected based on previous studies demonstrating CB2 receptor‐mediated protective effects in various organs (Çakır et al., 2019; Emamghoreishi et al., 2012; Farkhad et al., 2012; Mokhtari & Mohammadi, 2012; Muñoz‐Luque et al., 2008; Tutun & Baydan, 2018) (Figure 1).

### 2.6 Tissue removal and analysis

At the end of the 14‐day experimental period, the rats were anaesthetized by intraperitoneal administration of xylazine (5 mg/kg) and ketamine (45 mg/kg). Blood samples were collected by cardiac puncture before sacrifice. The animals were killed by exsanguination via terminal cardiac puncture under deep anaesthesia. Liver tissues were rapidly removed, and serum was separated from the collected blood for biochemical and histological analyses. Tissue samples taken for biochemical analysis were stored at −80°C. For histological analysis, liver tissue samples were fixed in 10% formaldehyde solution.

### 2.7 Biochemical assays

At the end of the study, approximately 2 mL of blood was collected by cardiac puncture from the animals and centrifuged at 4000 rpm (RCF: ~1790 × g) for 10 min. Serum samples were analysed in the Central Laboratory of Inonu University Turgut Ozal Medical Center with an autoanalyser using spectrophotometric methods to measure aspartate aminotransferase (AST), alanine aminotransferase (ALT), total protein (TP) and albumin levels (Arinola, 2014).

### 2.8 Measurement of hepatic GRP78, ATF4, CHOP, IL‐23, IL‐17, HSP72 and calpain levels by enzyme‐linked immunosorbent assay

Serum and liver tissue samples were removed from −80°C storage and thawed at 4°C before analysis. Liver tissues were weighed and homogenized in phosphate‐buffered saline. The resulting homogenates were analysed for GRP78, ATF4, CHOP, IL‐17, IL‐23, HSP72 and calpain levels using enzyme‐linked immunosorbent assay (ELISA) kits (Sunred Bio, Shanghai, China) according to the manufacturer's instructions. Serum samples were analysed for IL‐17 and IL‐23 levels using ELISA, as were liver tissue homogenates. Concentrations were determined from standard curves generated from optical density measurements. The standard curves were fitted by regression analysis using computer‐based curve‐fitting software, and analyte concentrations were calculated accordingly.

### 2.9 Histological and immunohistochemical analysis

For light microscopic evaluation, liver samples were fixed in 10% formalin. The liver samples were processed using routine histological techniques and embedded in paraffin. Paraffin‐embedded specimens were cut into 5‐µm‐thick sections, mounted on slides, and stained with hematoxylin and eosin (H&E). Tissue samples were examined using a Leica DFC280 light microscope and a Leica Q Win Image Analysis system (Leica Microsystems, Wetzlar, Germany).

For immunohistochemical (IHC) analysis, sections were mounted on polylysine‐coated slides. After rehydration, the sections were transferred to citrate buffer (pH 7.6) and heated in a microwave oven for 20 min. After cooling for 20 min at room temperature, the sections were washed with phosphate‐buffered saline (PBS). The sections were incubated in 0.3% H_2_O_2_ for 7 min and then washed with PBS. Sections were incubated with a primary rabbit polyclonal anti‐caspase‐3 antibody (PA1302, Boster Biological Technology, Pleasanton, CA, USA) for 2 h. They were then rinsed in PBS and incubated with biotinylated goat anti‐polyvalent antibody for 10 min and streptavidin peroxidase for 10 min at room temperature. Staining was completed using a chromogen and substrate for 15 min, followed by counterstaining with Mayer's haematoxylin for 1 min. The slides were then rinsed in tap water and dehydrated. The caspase‐3 antibody was used according to the manufacturer's instructions. Caspase‐3‐positive cells showed brown staining. Histopathological evaluation was performed in a blinded manner by an experienced histologist. Liver injury was assessed semi‐quantitatively according to Suzuki's criteria based on the degree of sinusoidal congestion, hepatocyte vacuolization and necrosis. Each parameter was graded on a scale of 0–4, and the total histopathological damage score was calculated for each sample (Suzuki et al., 1993).

### 2.10 Statistical analysis

Statistical analyses were performed using SPSS Statistics version 22.0 (IBM Corp., Armonk, NY, USA) and MedCalc (MedCalc Software Ltd, Ostend, Belgium) software. Normality of the data was assessed using the Shapiro–Wilk test. As the data were not normally distributed, differences among groups were analysed using the non‐parametric Kruskal–Wallis test, followed by a Bonferroni‐corrected Mann–Whitney *U*‐test for two‐groups comparisons. Changes in animal body weight before and after the experimental period were assessed using the Wilcoxon signed‐rank test. In accordance with the journal's statistical reporting policy, individual data points are available as supplementary information upon request (Bailey et al., 2023).

### 2.11 Statistical analysis of histology

Statistical analyses were made with SPSS 27.0 and MedCalc 11.0 statistical programs. All data are expressed as arithmetic means ± SD. For comparisons between groups, Kruskal–Wallis and Connover tests were used. Exact *P*‐values are given where available, and *P <* 0.0001 was accepted as statistically significant. Histopathological score is given in Table 5.

## 3 RESULTS

### 3.1 Changes in animal body weight

Within‐group analyses showed no significant change in body weight in CSG (*P =* 0.9650) and JG (*P =* 0.3640), whereas body weight decreased significantly in PG (*P =* 0.0010) and PJG (*P =* 0.0150) over the experimental period (Table 1).

**TABLE 1:** Changes in body weight at the beginning and end of the study.

	BW (g)		
	Pre‐experiment	Post‐experiment	*P*
**CSG**	452 (423–493)	453 (418–500)^a^	0.9650
**PG**	436 (379–478)	356(335–416)^b^	0.0010
**PJG**	453 (385–509)	403 (350–489)^ab^	0.0150
**JG**	440 (382–504)	428 (372–499)^a^	0.3640

*Note*: Data are presented as median (min–max). Superscript letters reproduce the original between‐group *post hoc* grouping; within‐group exact *P*‐values are shown in the final column. Bonferroni‐adjusted pairwise *P*‐values: CSG–PG: 0.0025; CSG–PJG: 0.0627; CSG–JG: 0.7239; PG–PJG: 0.0698; PG–JG: 0.0056; PJG–JG: 1.000.

### 3.2 Analysis of IL‐17 and IL‐23 levels in serum and liver tissue

No significant between‐group differences were observed in IL‐17 levels in liver tissue homogenates (*P =* 0.4800) or serum (*P =* 0.7730), or in IL‐23 levels in liver tissue homogenates (*P =* 0.4800) or serum (*P =* 0.2250) (Table 2).

**TABLE 2:** Results of IL‐17 and IL‐23 analyses.

		CSG (*n* = 9)	PG (*n* = 9)	PJG (*n* = 9)	JG (*n* = 10)	*P*
**IL‐17 (pg/ml)**	**Homogenate**	11.54 (0–26.46)	26.11 (0–62.6)	12.95 (0– 72.07)	9.70 (0– 21.72)	0.4800
	**Serum**	0.40 (0.23–1.38)	0.28 (0.19–0.34)	0.30 (0.23–0.38)	0.30 (0.24–0.36)	0.7730
**IL‐23 (pg/ml)**	**Homogenate**	1.19 (0– 2.78)	1.95 (0–2.87)	1.56 (0– 2.34)	1.07 (0.27–4.39)	0.4800
	**Serum**	0.13 (0.08–0.22)	0.13 (0.1–0.15)	0.15 (0.12–0.18)	0.14(0.13–0.16)	0.2250

*Note*: Data are presented as median (min–max).

### 3.3 Analysis of hepatic GRP78, CHOP, ATF4, calpain and HSP72 protein levels

No significant between‐group differences were observed in hepatic GRP78 (*P =* 0.4200), CHOP (*P =* 0.6600), ATF4 (*P =* 0.4200) or HSP72 (*P =* 0.2320) levels. In contrast, calpain levels differed significantly among the groups (*P =* 0.0017). Compared with CSG, calpain levels were significantly higher in PG (*P =* 0.0325) and PJG (*P =* 0.0076) following Bonferroni adjustment (Table 3).

**TABLE 3:** GRP78, CHOP, ATF4, Calpain and HSP72 analyses.

	CSG (*n* = 9)	PG (*n* = 9)	PJG (*n* = 9)	JG (*n* = 10)	*P*
**GRP78 (pg/ml)**	1.73 (1.64–2.44)	1.88 (1.49–2.11)	1.77 (1–2.19)	1.57 (1.31–2.6)	0.4200
**CHOP (pg/ml)**	1.5 (0–2.7)	1.1 (0– 1.68)	1.02 (0.12–2.17)	1.37 (0–3.31)	0.6600
**ATF4 (pg/ml)**	0.33 (0.16–0.57)	0.37 (0.16–0.56)	0.29 (0.06–0.56)	0.24 (0.15–0.49)	0.4200
**Calpain (pg/ml)**	0.21 (0.11–0.33) ^a^	0.28 (0.15–0.65) ^b^	0.35 (0.27–0.44) ^b^	0.23 (0.21–0.6) ^ab^	0.0017
**HSP72 (pg/ml)**	0.22 (0.11–0.32)	0.29 (0.15–0.48)	0.29 (0.19–0.38)	0.28 (0.17–0.39)	0.2320

*Note*: Data are presented as median (min–max). Different superscript letters indicate statistically significant between‐group differences. Bonferroni‐adjusted pairwise *P*‐values for calpain: CSG–PG: 0.0325; CSG–PJG: 0.0076; CSG–JG: 1.0000; PG–PJG: 1.0000; PG–JG: 0.4350; PJG–JG: 0.0540.

### 3.4 Analysis of blood ALT, AST, albumin, and total protein levels

Significant between‐group differences were observed in serum ALT, AST, albumin and total protein levels (all *P <* 0.0001) (Table 4). Compared with CSG, PG and PJG had lower ALT (*P =* 0.0021 and *P =* 0.0021, respectively), AST (*P =* 0.0021 for both), albumin (*P =* 0.0031 and *P =* 0.0016, respectively) and total protein (*P =* 0.0056 and *P =* 0.0017, respectively). Compared with JG, PG and PJG also had lower ALT (*P =* 0.0026 and *P =* 0.0014, respectively), AST (*P =* 0.0020 and *P =* 0.0014, respectively), albumin (*P =* 0.0210 and *P =* 0.0117, respectively) and total protein (*P =* 0.0217 and *P =* 0.0053, respectively).

**TABLE 4:** Results of ALT, AST, albumin and total protein (TP) analyses.

	CSG (*n* = 9)	PG (*n* = 9)	PJG (*n* = 9)	JG (*n* = 10)	*P*
**ALT (U/L)**	63.7 (55–72) ^a^	20 (14– 51) ^b^	26 (13–35) ^b^	57 (46–167) ^a^	<0.0001
**AST (U/L)**	99 (80–120) ^c^	48 (39– 73) ^d^	49 (36– 64) ^d^	87 (71–202) ^c^	<0.0001
**Albumin (g/dL)**	1.1 (1.00–1.20) ^e^	0.8 (0.70–1.00) ^f^	0.8 (0.7–0.9) ^f^	0.99 (0.80–1.10) ^e^	<0.0001
**TP (g/dL)**	6.1 (5.70–6.30) ^g^	5.4 (5.10–6.20) ^h^	5.21(4.6–5.5) ^h^	6 (5.40–6.50) ^g^	<0.0001

*Note*: Data are presented as median (min–max). Different superscript letters indicate statistically significant between‐group differences. Bonferroni‐adjusted pairwise *P*‐values: ALT: CSG–PG: 0.0021; CSG–PJG: 0.0021; CSG–JG: 1.0000; PG–PJG: 1.0000; PG–JG: 0.0026; PJG–JG: 0.0014; AST: CSG–PG: 0.0021; CSG–PJG: 0.0021; CSG–JG: 1.0000; PG–PJG: 1.0000; PG–JG: 0.0020; PJG–JG: 0.0014; albumin: CSG–PG: 0.0031; CSG–PJG: 0.0016; CSG–JG: 0.5850; PG–PJG: 1.0000; PG–JG: 0.0210; PJG–JG: 0.0117; TP: CSG–PG: 0.0056; CSG–PJG: 0.0017; CSG–JG: 1.0000; PG–PJG: 1.0000; PG–JG: 0.0217; PJG–JG: 0.0053.

### 3.5 Histological results

**FIGURE 2:**
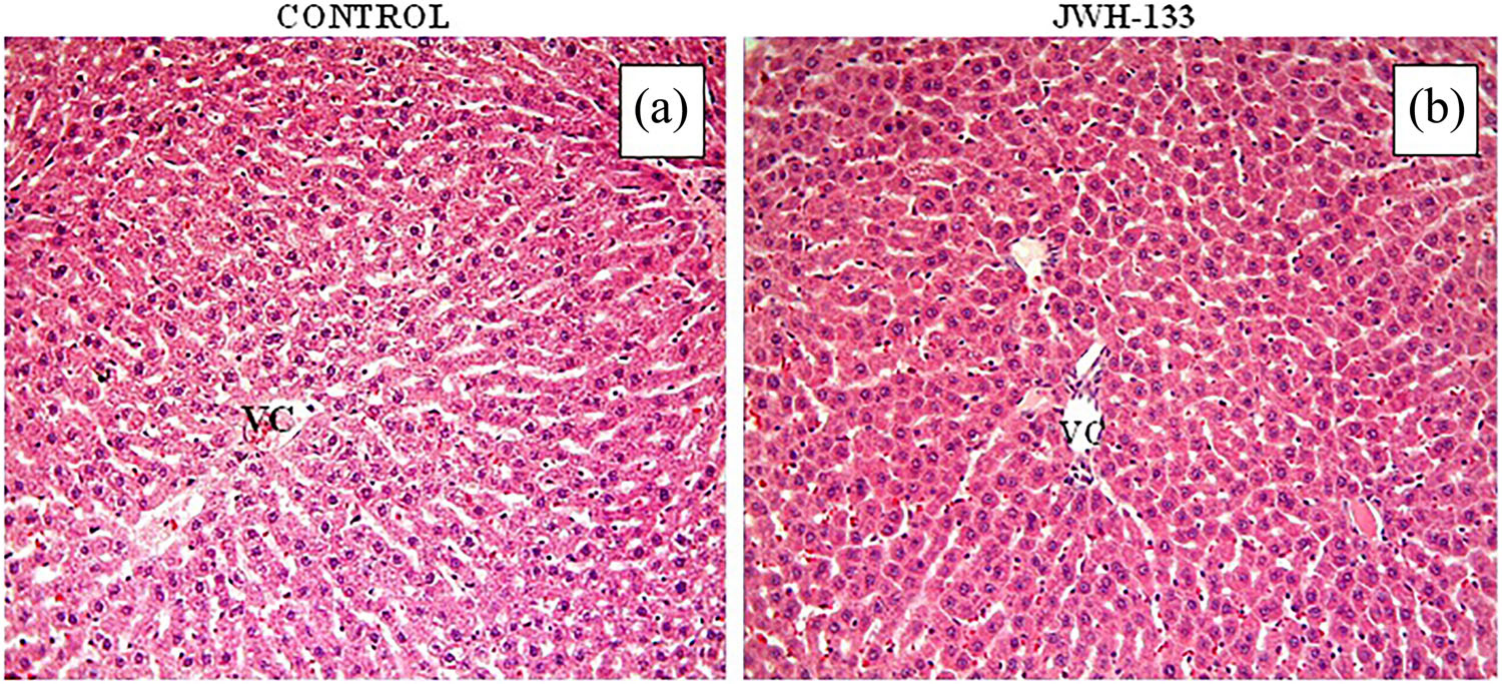
In the control group (CSG) (a) and JWH‐133 group (JG) (b), liver tissue exhibited a normal histological appearance. Haematoxylin and eosin staining, magnification ×20. VC, vena centralis.

Histopathological damage scores were significantly higher in PG than in CSG, PJG and JG (all *P <* 0.0001) (Table 5). In CSG and JG, the liver tissues showed normal histological appearance (Figure 2).

**TABLE 5:** Histopathological damage scores among the groups.

Groups	Histopathological damage
**CSG (*n* = 9)**	0.56 ± 0.24^a^
**PG (*n* = 9)**	2.37 ± 0.24^b^
**PJG (*n* = 9)**	1.33 ± 0.33^c^
**JG (*n* = 10)**	0.97 ± 0.32^a^

*Note*: Data are presented as means ± SD. Superscript letters reproduce the original *post hoc* grouping; comparisons of PG with CSG, PJG and JG were all *P <* 0.0001.

**FIGURE 3:**
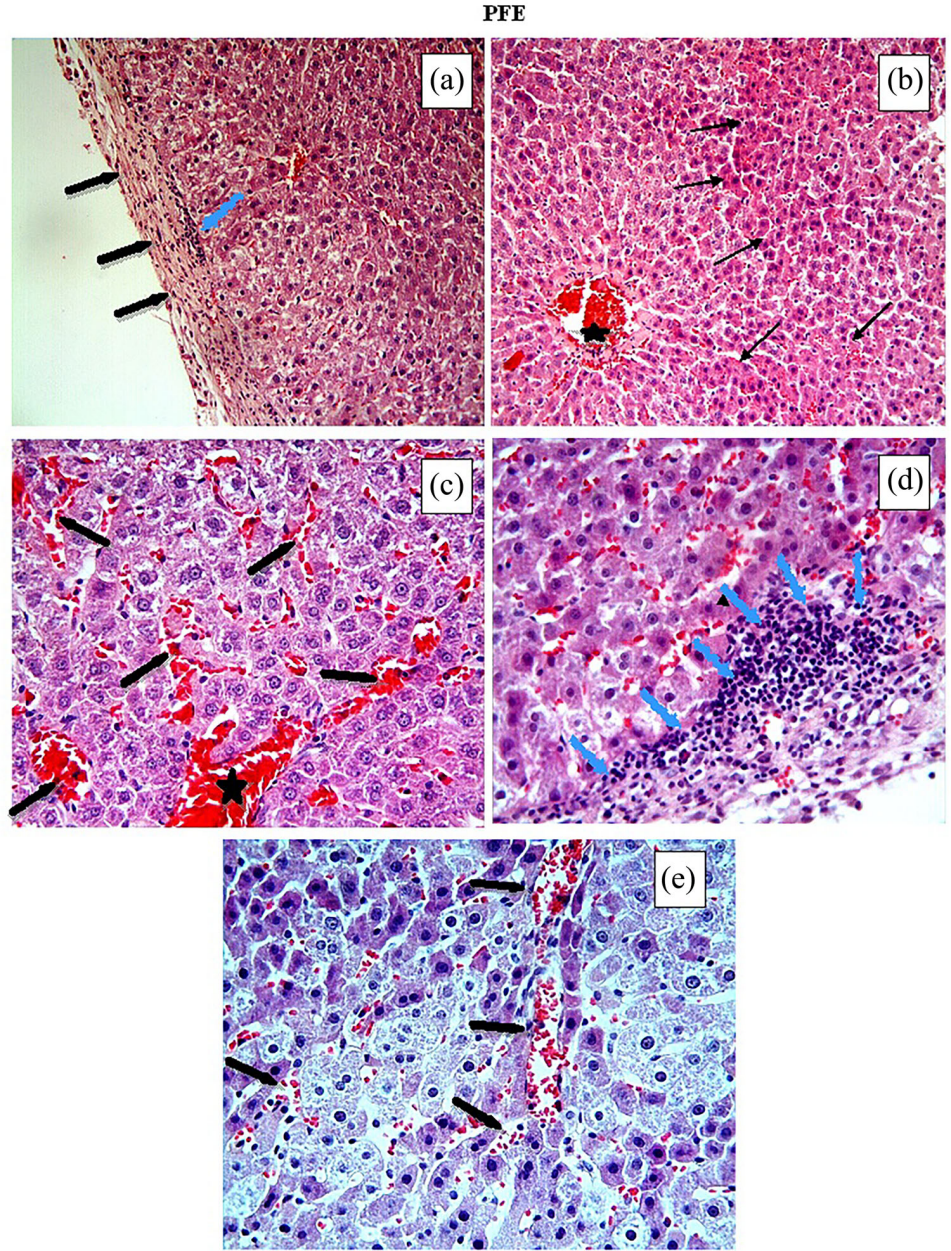
In the PFE group (PG), thickening of the Glisson capsule (thick black arrow) and mononuclear cell infiltration (blue arrows) (a, d), eosinophilic stained pyknotic nuclei cells (thin black arrows) (b), vascular congestion (black asterisks) (c), haemorrhage (thick black arrows) (c, e), and swelling of hepatocytes (e) were observed. Haematoxylin and eosin staining, magnification ×20 (a, b) and ×40 (c–e).

**FIGURE 4:**
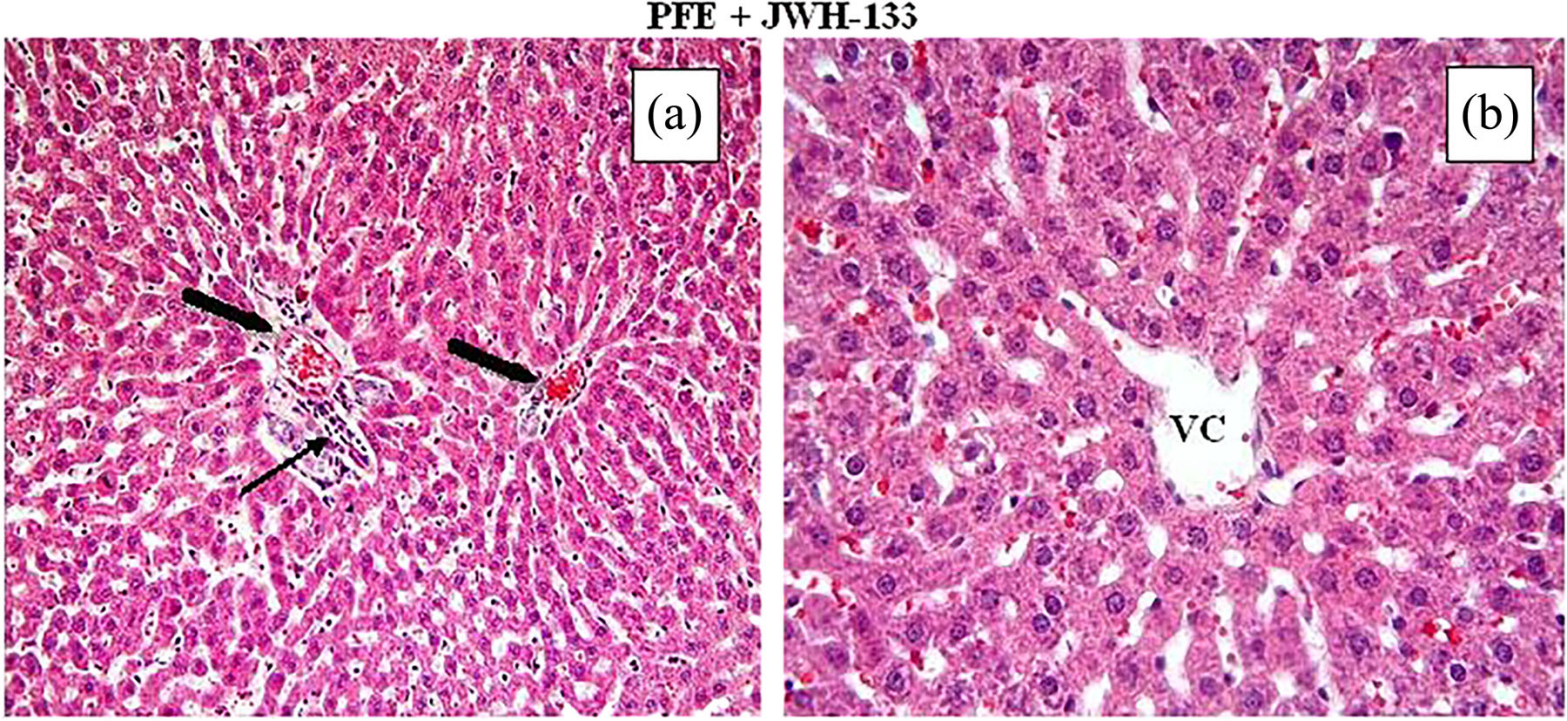
Representative histopathological findings in the PFE + JWH‐133 group (PJG). Little vascular congestion (thick black arrows) (a), mononuclear cell infiltration (thin black arrows) (a), and sinusoidal dilatation (b) was observed in this group. Haematoxylin and eosin staining, magnification ×20 (a) and ×40 (b). VC, vena centralis.

In liver tissue samples from PG, thickening (black thick arrow) and infiltration (white arrow) in the Glisson capsule (Figure 3a), vascular congestion (Figure 3b,c), eosinophilic stained pyknotic nuclei cells (black thin arrows) (Figure 3b,e), haemorrhage (black thick arrows) (Figure 3c,e), mononuclear cell infiltration (black thin arrows) (Figure 3d) and swelling of hepatocytes (Figure 3e) were observed. Histopathological damage was significantly lower in PJG than in PG (*P <* 0.0001). In PJG, little vascular congestion (black thick arrows) (Figure 4a), mononuclear cell infiltration (black thin arrows) and sinusoidal dilatation (Figure 4b) was observed.

### 3.6 Immunohistochemical results

**FIGURE 5:**
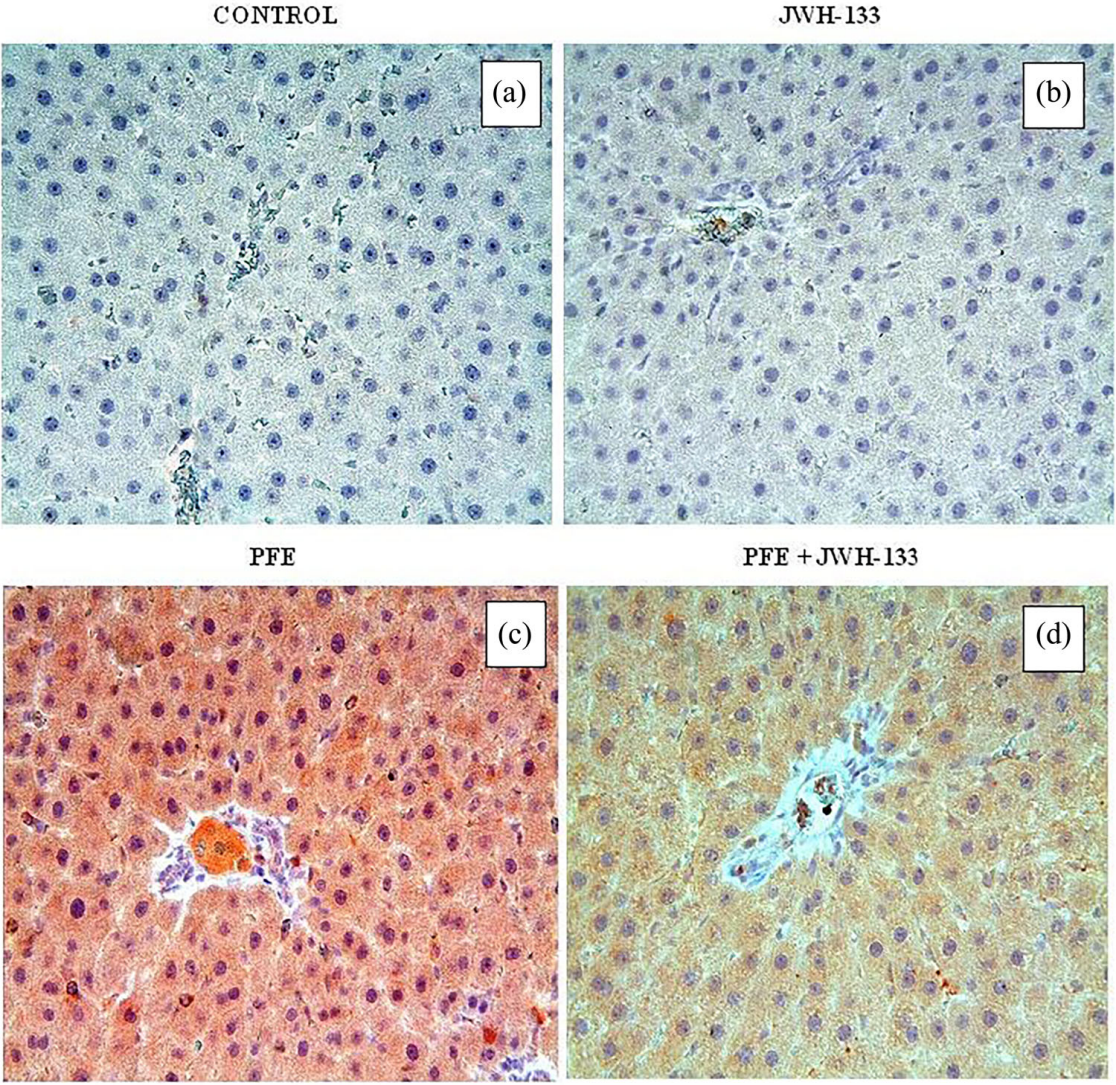
Caspase‐3 immunostaining for all groups. (a, b) Immunohistochemical expression of caspase‐3 in the control group (CSG) (a) and JWH‐133 group (JG) (b). (c) The number of caspase‐3 positive cells was high in the PFE group (PG). (d) The proportion of immunohistochemical caspase‐3 positively stained cells was minimal in the PFE + JWH‐133 group (PJG). Magnification ×40 (a–d).

Immunohistochemical caspase‐3 positively stained cells were not observed in CSG (Figure 5a) and JG (Figure 5b). The number of caspase‐3 positive cells was high in PG (Figure 5c). The proportion of immunohistochemical caspase‐3 positively stained cells was minimal in PJG (Figure 5d).

## 4 DISCUSSION

In the present study, rats treated with PFE exhibited greater weight loss compared to animals receiving PFE in combination with the cannabinoid type 2 receptor agonist JWH‐133. Farokhi et al. (2012) demonstrated that PF extract administered for 4 weeks induced weight loss in diabetic rats. The liver is the primary target organ of xenobiotic stress due to its role in xenobiotic metabolism and its involvement in the gastrointestinal system. In addition, pathological and histological evaluations showed severe liver damage in animals injected with PFE. These findings suggest that liver damage caused by PFE may impair hepatic metabolic functions, thereby contributing to weight loss in animals. Another notable finding was that weight loss in animals receiving PFE was partially attenuated by JWH‐133 administration.

Dursun et al. (2019) reported that acute liver toxicity developed in patients with a significant increase in ALT and AST levels after PF consumption. Mokhtari & Mohammadi (2012) demonstrated that ALT and AST levels were elevated in rats administered PF extract for 3 weeks compared to other rats. In addition, Shen et al. (2019) demonstrated that osthole induced cytotoxicity and apoptosis in human normal liver L02 cells and activated oxidative stress‐ and ER stress‐related pathways. On the other hand, a study conducted by Farokhi et al. (2012) on diabetic rats found that ALT and AST levels decreased in rats treated with hydroalcoholic PFE daily for 4 weeks, indicating that the biochemical response may be modulated by both treatment duration and underlying metabolic state. In the present study, despite clear histopathological evidence of hepatocellular injury, haemorrhage, mononuclear infiltration, and increased caspase‐3 immunoreactivity, serum ALT and AST levels in PG and PJG were unexpectedly lower than those in CSG and JG. Several mechanisms may account for this apparent discrepancy. First, serum aminotransferase activity reflects enzyme release from damaged but still viable hepatocytes; in advanced injury characterized by extensive necrosis and apoptosis, the pool of hepatocytes capable of releasing these enzymes may be reduced, resulting in paradoxically low circulating levels (Craig et al., 1991; Haber et al., 1995). The concurrent reductions in serum albumin and total protein observed in PG and PJG support this interpretation, as decreased synthetic capacity is a hallmark of widespread hepatic dysfunction (Huang et al., 2020). Second, ALT and AST follow a characteristic temporal profile in toxic hepatic injury, typically peaking during the early acute phase and declining thereafter even when histological damage persists or progresses (McGill, 2016). Because biochemical sampling in our study was performed only on day 14, an earlier peak in transaminase activity may have been missed; the absence of an interim time point is therefore a limitation of the present design. Third, sustained intraperitoneal administration of a hydroalcoholic plant extract differs substantially from the single high‐dose oral exposure typically responsible for the acute clinical presentation, and may engage adaptive or compensatory responses that further alter the biochemical profile. Taken together, these considerations indicate that serum aminotransferase levels in chronic experimental models of plant‐induced hepatotoxicity should be interpreted in conjunction with histological and synthetic‐function markers rather than in isolation. The combined pattern of marked histopathological injury increased apoptotic activity, and reduced hepatic synthetic capacity in our study is consistent with substantial PF‐induced liver damage despite the lower serum transaminase values.

It has been demonstrated that elevated levels of the cytokines IL‐17 and IL‐23 can stimulate the inflammatory response and lead to liver damage (Meng et al., 2012). The liver, which is a crucial organ for drug biotransformation, is at significant risk for herbal hepatotoxicity. Certain plant compounds may cause hepatotoxic effects through pathogenic mechanisms that induce apoptosis, including endoplasmic reticulum (ER) stress, cytochrome P450 bioactivation, oxidative stress and mitochondrial damage (Yan et al., 2023). Although IL‐17 and IL‐23 levels were expected to increase in response to liver injury, no significant differences were observed among the groups in the present study.

Currently, plasma total protein and albumin levels are frequently evaluated to diagnose liver damage. Low total protein and albumin levels indicate decreased function and widespread damage to the liver, which is responsible for their production (Huang et al., 2020). Similarly, in our study, serum total protein and albumin levels were lower in both PG and PJG than in CSG and JG, whereas PG and PJG did not differ from each other. These findings were consistent with the histopathological evidence of diffuse liver injury observed in PG.

The endocannabinoid system is a promising target for treating various inflammatory diseases, including liver diseases (Gabbay et al., 2005). Avraham et al. (2008) have shown that the CB2 agonist HU‐308 reduced elevated serum AST and ALT levels in mice with thioacetamide‐induced acute liver failure. Moreover, it has been reported that AST and ALT levels decreased when the CB2 agonists JWH‐133 and HU‐910 were used to treat I/R injury (Bátkai et al., 2007; Horváth et al., 2012). In carbon tetrachloride (CCl_4_)‐induced liver injury, a CB2 agonist has been shown to reduce increased serum AST and ALT levels (Teixeira‐Clerc et al., 2010). Çakır et al. (2019) have demonstrated that JWH‐133 administration reduced the elevated caspase‐3 levels resulting from okadaic acid‐induced damage in rat brain tissue. In our study, although JWH‐133 significantly attenuated histopathological liver damage induced by PFE, serum ALT and AST levels were not significantly lower than those in PG.

Shen et al. (2019) demonstrated that osthole, a coumarin found in PF, induced apoptosis in human normal liver L02 cells in association with activation of ER stress signalling. Our study aimed to examine PFE‐induced liver damage in relation to ER stress and to evaluate the protective effect of JWH‐133 against this damage. In the present study, no significant changes were observed in hepatic CHOP, GRP78 or ATF4 levels following PFE administration. These findings suggest that ER stress pathways may not play a dominant role in PF‐induced hepatotoxicity under the experimental conditions used in this study. Several factors may explain why PF‐induced hepatotoxicity appeared to proceed largely independently of canonical ER stress signalling under our conditions. The injury may be driven predominantly by calcium‐dependent and oxidative‐stress pathways. This view is supported by the elevated calpain levels observed here. Such pathways can operate independently of the canonical PERK/ATF4/CHOP, IRE1 and ATF6 arms of the unfolded protein response. Activation of these ER stress markers is also frequently transient. It may therefore have peaked before the single day‐14 sampling point. Consequently, an earlier or compartment‐specific response could have been missed. In addition, ELISA quantification in whole‐tissue homogenates has limited sensitivity. This approach may not detect localized or short‐lived changes in unfolded protein response components. Finally, the coumarin constituents of PF may promote hepatocyte apoptosis mainly through mitochondrial and caspase‐dependent routes. These routes do not necessarily require sustained canonical ER stress signalling.

Xie et al. (2020) have reported that dithiothreitol, an ER stress inducer, can stimulate the activation of caspase‐12 and induce apoptosis in hepatocytes in vitro. This may occur through an increase in intracellular Ca^2+^ levels and increased calpain‐2 expression in hepatocytes. Additionally, it was found that suppressing calpain‐2 expression could attenuate caspase‐12 cleavage and subsequent apoptosis (Xie et al., 2020). Calpain has been shown to modulate various pro‐apoptotic and anti‐apoptotic molecules, including caspases, Bax, Bid, NF‐κB, p53, Bcl‐2 and Bcl‐xL (Daniel, 2000). A study by Jing et al. (2012) demonstrated that calpain activation leads to oxidative stress in cells, and that calpain inhibition reduces oxidative stress. Our analysis of calpain levels among the groups showed that calpain levels in PG and PJG were significantly higher. In this study, ER stress markers did not increase significantly in tissue homogenates as measured by ELISA. The increase in calpain levels despite the absence of significant ER stress marker activation may indicate the involvement of calcium‐dependent or oxidative stress‐related pathways in PFE‐induced hepatotoxicity. HSP levels increase under conditions such as hypoxia, oxidative stress, ER stress, acidosis and an increase in intracellular calcium. They perform several functions, including facilitating the degradation of misfolded proteins, acting as chaperones, and protecting correctly synthesized proteins from proteases. HSP production increases in response to ER stress, thereby protecting the cell (Topal et al., 2021). Consistent with the results for ER stress markers, HSP72 levels also did not differ significantly among the groups.

Shokoohinia et al. (2014) have shown that, in vitro, osthole, a coumarin isolated from the PF plant, increases the levels of caspase‐3, caspase‐8 and caspase‐9, which induce tissue damage by activating apoptotic pathways. Additionally, Shen et al. (2019) have reported that, in vitro, osthole caused cell damage by increasing ER stress and oxidative stress, as well as by activating caspase‐3 and caspase‐9, thereby triggering apoptotic pathways. Our findings are consistent with previous studies demonstrating the pro‐apoptotic effects of osthole and PF‐derived compounds.

Researchers have demonstrated that CB2 receptor agonists protect against hepatic damage by reducing endothelial cell activation, the expression of adhesion molecules and inflammatory cytokines, and the adhesion of inflammatory cells (Bátkai et al., 2007; Rajesh et al., 2007). Recent studies on the liver have shown that Kupffer cells and fibrogenic liver cells are stimulated after acute and chronic tissue damage, and CB2 receptor expression in these cells increases (Julien et al., 2005; Mallat et al., 2007; Teixeira‐Clerc et al., 2010). Activation of CB2 receptors in hepatic myofibroblasts has been associated with antifibrogenic effects, while CB2 agonism reduced hepatocyte apoptosis and promoted liver regeneration (Teixeira‐Clerc et al., 2010). Our results showed that the CB2 receptor agonist JWH‐133 reduced diffuse liver tissue necrosis and significantly improved histopathological findings in animals receiving PFE.

### 4.1 Limitations

Several limitations should be acknowledged. First, only 14‐day intraperitoneal PF administration with once‐daily dosing and a single endpoint was evaluated; therefore, dose–response and time‐course relationships, including possible transient aminotransferase elevations, could not be assessed. Second, only male rats were included, limiting conclusions regarding sex‐dependent effects. Third, CB2 involvement was inferred pharmacologically, and co‐administration of a CB2‐selective antagonist such as AM630 would be required to establish receptor specificity. Fourth, ER stress, calpain and HSP72 were assessed by ELISA without orthogonal validation by western blotting or qRT‐PCR, potentially limiting detection of transient or compartment‐specific changes. Fifth, caspase‐3 immunoreactivity was evaluated semi‐quantitatively rather than by formal cell counting. Finally, the study did not include a non‐injected naïve control group or cohorts with longer follow‐up periods. In addition, a detailed mechanistic investigation of how JWH‐133 attenuates PF‐induced damage was beyond the scope of the present study. These limitations highlight important directions for future research.

### 4.2 Conclusion

In conclusion, PFE induced substantial hepatic injury in rats, characterized by marked histopathological damage, reduced hepatic synthetic function, increased calpain levels and enhanced caspase‐3 immunoreactivity. Notably, although serum ALT and AST were paradoxically lower in the PFE‐treated groups, this pattern, together with the concurrent reductions in serum albumin and total protein, is best interpreted as reflecting advanced hepatocellular loss and reduced hepatic synthetic capacity rather than milder injury. The lack of significant changes in CHOP, GRP78, ATF4, HSP72, IL‐17 and IL‐23 suggests that sustained canonical ER stress and these inflammatory pathways may not predominate under the present experimental conditions. In contrast, increased calpain and caspase‐3 expression may indicate the involvement of calcium‐dependent, oxidative stress‐related and apoptotic mechanisms. JWH‐133 attenuated PFE‐induced histopathological injury and partially limited body weight loss, supporting a potential hepatoprotective role of CB2 receptor activation, although this effect was not reflected by serum aminotransferase levels. Further studies incorporating time‐course analyses and complementary oxidative stress, apoptosis and CB2‐related molecular markers are needed to clarify the underlying mechanisms and therapeutic potential of CB2 receptor agonists in PF‐associated hepatotoxicity.

## AUTHOR CONTRIBUTIONS

Conception or design of the work: Mesut Çelik, Aslı Çetin Taşlıdere, Zeynal Mete Karaca, Halil Düzova, İbrahim Halil Geçibesler, Ali Rıza Çalışkan and Çağatay Taşkapan. Acquisition, analysis or interpretation of data: Mesut Çelik, Aslı Çetin Taşlıdere, Zeynal Mete Karaca, İbrahim Halil Geçibesler, Çağatay Taşkapan and Kübranur Korkmaz. Drafting the manuscript: Mesut Çelik, Halil Düzova, Ali Rıza Çalışkan and Kübranur Korkmaz. Revising the manuscript critically for important intellectual content: Mesut Çelik, Halil Düzova and Kübranur Korkmaz. All authors approved the final version of the manuscript and agree to be accountable for all aspects of the work in ensuring that questions related to the accuracy or integrity of any part of the work are appropriately investigated and resolved. All persons designated as authors qualify for authorship, and all those who qualify for authorship are listed.

## CONFLICT OF INTEREST

None declared.

## GENERATIVE AI STATEMENT

All authors acknowledge that during the preparation of this manuscript, Grammarly and generative AI tools were used solely for spelling, grammar, language editing, and formatting purposes; no generative AI tools were used to generate scientific data, analyses, interpretations, or conclusions.

## Data Availability

The data presented in this study are available on request from the corresponding author.
